# Supplementary material for: Spatial Aspects of Gardens Drive Ranging in Urban Foxes (Vulpes vulpes): The Resource Dispersion Hypothesis Revisited
Source: Animals (Basel). 2020 Jul 9;10(7):1167. doi: 10.3390/ani10071167 (PMC7401560; doi:10.3390/ani10071167)
Supplement: Supplementary file 1 [file animals-10-01167-s001.zip › animals-771497 suppl -xml/Supplementary table 1.docx]

| **Fox ID** | **Weight (kg)** | **Tooth wear** | **Body Condition** | **Breeding status** |
| --- | --- | --- | --- | --- |
| A1 | 5.7 | Low | Very good | Testes scrotal |
| A2 | 6.4 | Low | Very good | Non-breeding (pre-breeding) |
| A3 | 6 | High | Good | Non-breeding (bred this year) |
| A4 | 6 | Low | Good | Lactating (enlarged nipples) |
| A5 | 5.7 | Low | Good | Lactating (enlarged nipples) |
| A6 | 6.3 | Medium | Good | Testes scrotal |
| A7 | 8.0 | Medium | Good | Testes scrotal |
| A8 | 8.8 | Low | Good | Testes scrotal |
| A9 | 5.2 | Low | Good | Non-breeding (pre-breeding) |
| A10 | 6.6 | Low | Very good | Testes abdominal (pre-breeding) |
| B1 | 6.4 | Medium | Very good | - |
| B2 | 7.5 | Medium | Very good | Testes scrotal |
| B3 | 6.4 | Medium | Good | - |
| B4 | 7.1 | Medium | Good | Testes scrotal |
| B5 | 6.5 | Low | Very good | Testes abdominal |
| B6 | 5.0 | High | Good | Non-breeding |
| B7 | 7.4 | Low | Very good | Testes scrotal |
| B8 | 6.4 | Low | Very good | Testes scrotal |
| B9 | 6.3 | Low | Good | Non-breeding |
| B10 | 5.8 | Medium | Good | Non-breeding (bred this year) |

**Table S1** : Variables used to estimate fox age and social status
