# Supplementary material for: Spatial Aspects of Gardens Drive Ranging in Urban Foxes (Vulpes vulpes): The Resource Dispersion Hypothesis Revisited
Source: Animals (Basel). 2020 Jul 9;10(7):1167. doi: 10.3390/ani10071167 (PMC7401560; doi:10.3390/ani10071167)
Supplement: Supplementary file 1 [file animals-10-01167-s001.zip › animals-771497 suppl -xml/Supplementary table 2.docx]

| **Explanatory variable** | **ꞵ coefficient (parameter estimate)** | **SE (ꞵ)** | ***t*** | ***P*** |
| --- | --- | --- | --- | --- |
| **Number of patches (garden scale)** | **0.57947** | **0.06963** | **8.322** | **0.000000014***** |
| **Edge density (garden scale)** | **0.00002** | **0.000008** | **2.134** | **0.047*** |
| **Number of patches**  **(landscape scale)** | **0.00428** | **0.00067** | **6.35** | **0.000001***** |
| **Garden size** | **-0.00144** | **0.00062** | **-2.325** | **0.032*** |
| **Ratio of gardens to combined other habitats** | **-0.12119** | **0.04556** | **-2.66** | **0.016**** |
| Year: |  |  |  |  |
| 2012 vs. 2013 | 0.12604 | 0.19071 | 0.661 | 0.518 |
| 2012 vs. 2014 | -0.02951 | 0.27484 | -0.107 | 0.916 |
| 2012 vs. 2015 | 0.17296 | 0.21289 | 0.812 | 0.428 |
| 2013 vs. 2014 | -0.15555 | 0.24809 | -0.627 | 0.540 |
| 2013 vs. 2015 | 0.04692 | 0.17702 | 0.265 | 0.794 |
| 2014 vs. 2015 | 0.20248 | 0.26553 | 0.763 | 0.428 |
| Season: |  |  |  |  |
| Summer vs. spring | 0.26317 | 0.17777 | 1.480 | 0.158 |
| Spring vs. autumn | 0.28299 | 0.17777 | 1.592 | 0.131 |
| Autumn vs. winter | 0.17115 | 0.22595 | 0.757 | 0.460 |
| Spring vs. winter | -0.11185 | 0.19722 | -0.567 | 0.579 |
| Summer vs. autumn | 0.01982 | 0.20918 | 0.095 | 0.926 |
| Summer vs. winter | 0.15132 | 0.22595 | 0.670 | 0.513 |
| Sex (male vs. female) | -0.1847 | 0.1341 | -1.378 | 0.185 |
| Housing density (HD) | 0.01478 | 0.0157 | 0.941 | 0.3601 |
| HD² | -0.00013 | 0.00017 | -0.787 | 0.4421 |
| Edge density (landscape scale) | 0.00027 | 0.00039 | 0.686 | 0.5012 |

**Table S2** Coefficients of the full list of variables separately modelled in univariate tests with core area size as the response in each case.
