# Supplementary material for: Spatial Aspects of Gardens Drive Ranging in Urban Foxes (Vulpes vulpes): The Resource Dispersion Hypothesis Revisited
Source: Animals (Basel). 2020 Jul 9;10(7):1167. doi: 10.3390/ani10071167 (PMC7401560; doi:10.3390/ani10071167)
Supplement: Supplementary file 1 [file animals-10-01167-s001.zip › Supplementary Figure 1.docx]

**
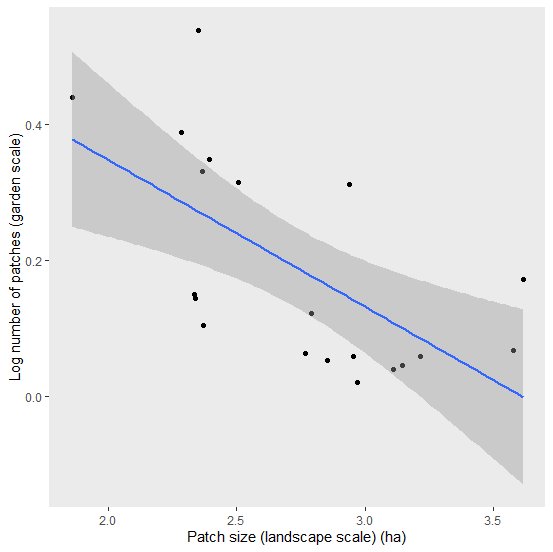

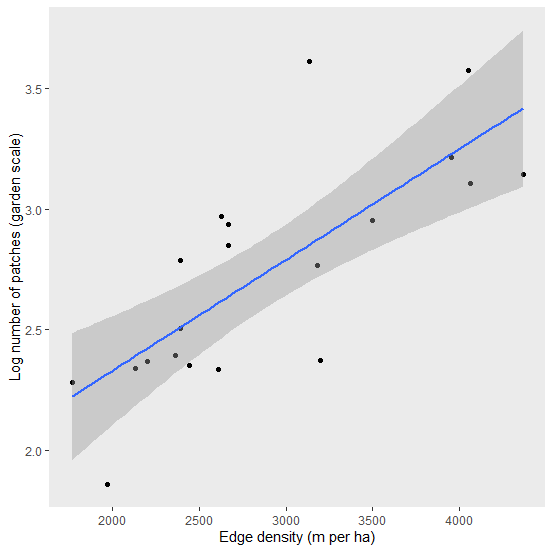
**

ꞵ=0.00005; SE = 0.0000094; *t*=4.93; *p < 0.001; adj R²=*0.55

ꞵ= -0.216; SE =0.062; *t*= -3.47; *p < 0.01; adj R²=*0.37

**
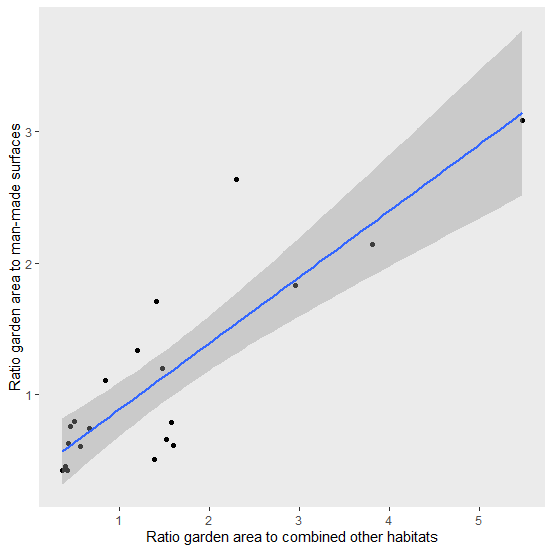

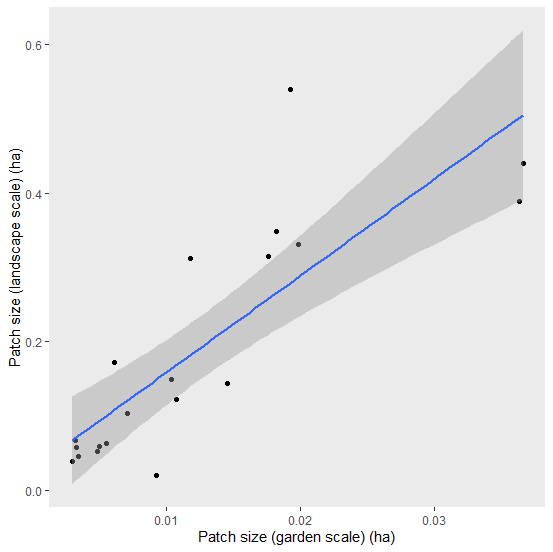
**

ꞵ=0.50; SE = 0.07; *t*=7.08; *p « 0.001; adj R²=*0.72

ꞵ=0.053; SE = 0.008; *t*=6.27; *p « 0.001; adj R²=*0.67

**
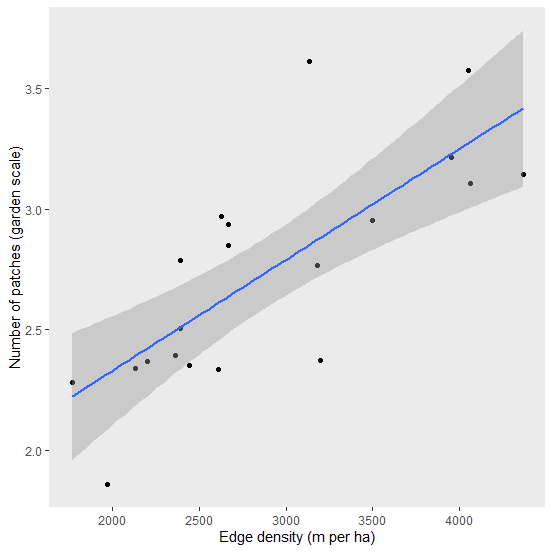

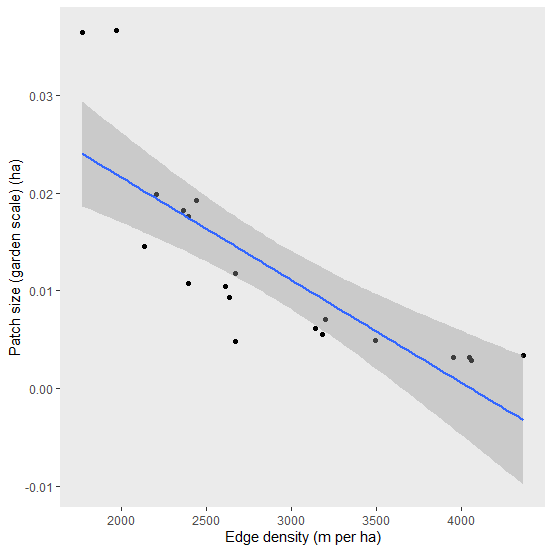
**

ꞵ = 0.000001; SE = 0.0000002; *t*= 4.93; *p < 0.001; adj R²=*0.55

ꞵ = -0.000001; SE = 0.0000002; *t*= -5.54; *p « 0.001; adj R²=*0.61

**
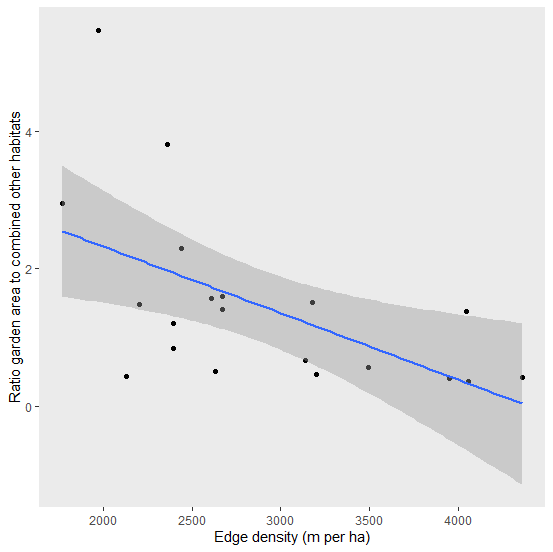

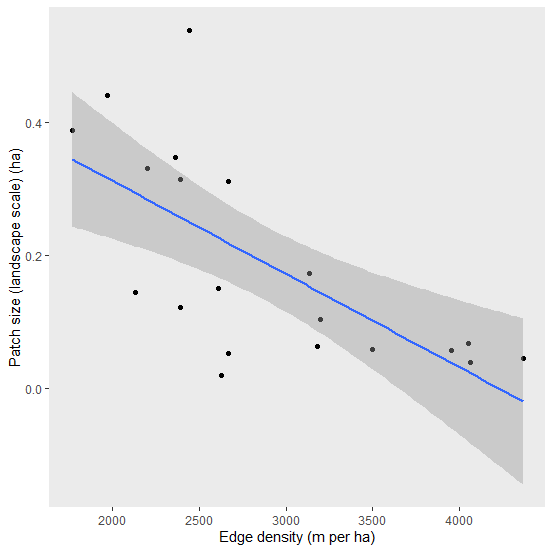
**

ꞵ = -0.000014; SE = 0.000004; *t*= -3.90; *p < 0.01; adj R²=*0.43

ꞵ = -0.0010; SE = 0.0003; *t*= -2.87; *p < 0.05; adj R²=*0.28

**
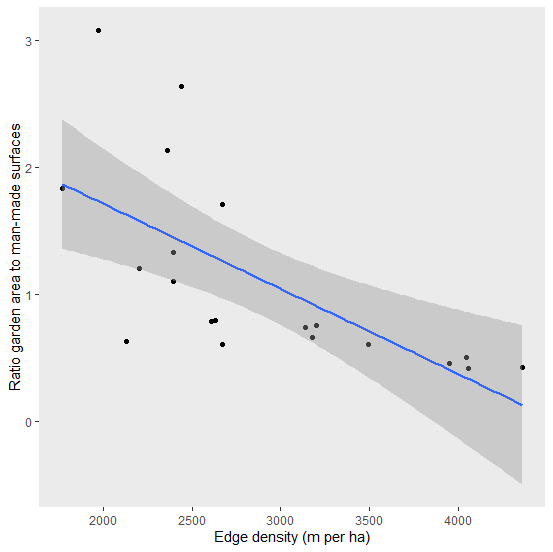

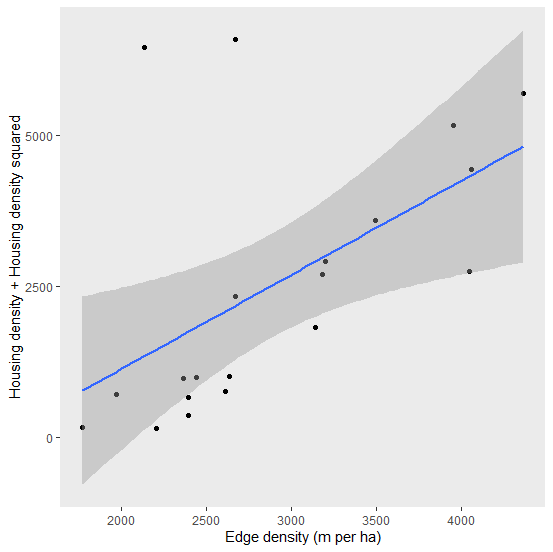
**

ꞵ = 1.558; SE = 0.554; *t*= 2.81; *p < 0.05; adj R²=*0.27

ꞵ = -0.0007; SE = 0.0002; *t*= -3.71; *p < 0.01; adj R²=*0.40

**
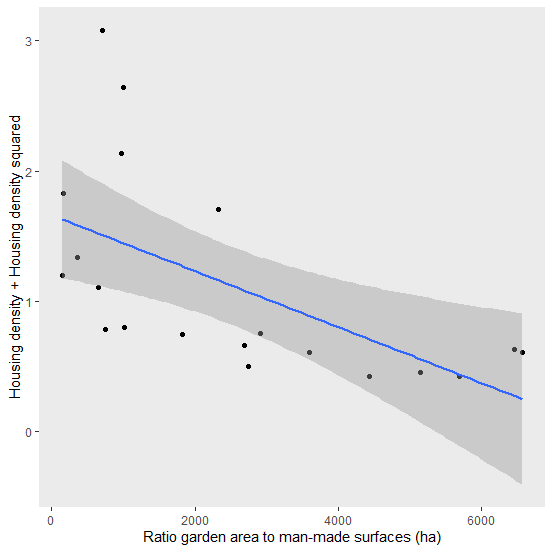

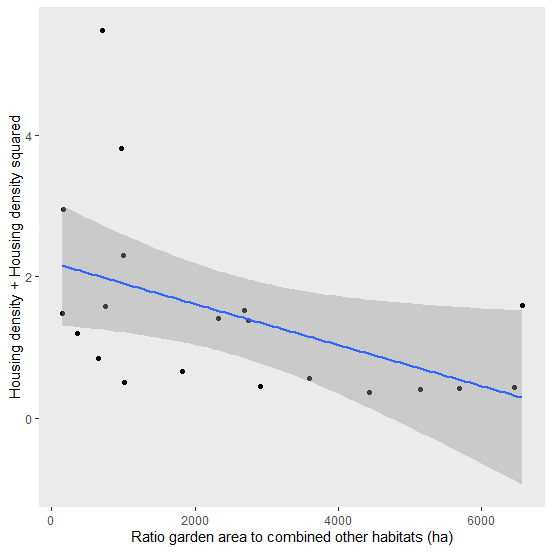
**

ꞵ = -1648.6; SE = 525.0; *t*= -3.140; *p < 0.05; adj R²=*0.32

ꞵ = -766.4; SE = 338.2; *t*= -2.27; *p < 0.05; adj R²=*0.18

**SI Figure 1** Collinearity plots for explanatory variables; showing coefficients in legends.
